# Exome-wide benchmark of difficult-to-sequence regions using short-read next-generation DNA sequencing

**DOI:** 10.1093/nar/gkad1140

**Published:** 2023-11-28

**Authors:** Atsushi Hijikata, Mikita Suyama, Shingo Kikugawa, Ryo Matoba, Takuya Naruto, Yumi Enomoto, Kenji Kurosawa, Naoki Harada, Kumiko Yanagi, Tadashi Kaname, Keisuke Miyako, Masaki Takazawa, Hideo Sasai, Junichi Hosokawa, Sakae Itoga, Tomomi Yamaguchi, Tomoki Kosho, Keiko Matsubara, Yoko Kuroki, Maki Fukami, Kaori Adachi, Eiji Nanba, Naomi Tsuchida, Yuri Uchiyama, Naomichi Matsumoto, Kunihiro Nishimura, Osamu Ohara

**Affiliations:** Laboratory of Computational Genomics, School of Life Sciences, Tokyo University of Pharmacy and Life Sciences, Hachioji, Tokyo 192-0392, Japan; Division of Bioinformatics, Medical Institute of Bioregulation, Kyushu University, Higashi-ku, Fukuoka 812-8582, Japan; DNA Chip Research Inc., Minato-ku, Tokyo 105-0022, Japan; DNA Chip Research Inc., Minato-ku, Tokyo 105-0022, Japan; Clinical Research Institute, Kanagawa Children's Medical Center, Minami-ku, Yokohama, Kanagawa 232-0066, Japan; Clinical Research Institute, Kanagawa Children's Medical Center, Minami-ku, Yokohama, Kanagawa 232-0066, Japan; Clinical Research Institute, Kanagawa Children's Medical Center, Minami-ku, Yokohama, Kanagawa 232-0066, Japan; Division of Medical Genetics, Kanagawa Children's Medical Center, Minami-ku, Yokohama, Kanagawa 232-0066, Japan; Department of Fundamental Cell Technology, Center for iPS Cell Research and Application (CiRA), Kyoto University, Sakyo-ku, Kyoto 606-8507, Japan; Department of Genome Medicine, National Center for Child Health and Development, Setagaya-ku, Tokyo 157-8535, Japan; Department of Genome Medicine, National Center for Child Health and Development, Setagaya-ku, Tokyo 157-8535, Japan; Department of Applied Genomics, Kazusa DNA Research Institute, Kisarazu, Chiba 292-0818, Japan; Department of Applied Genomics, Kazusa DNA Research Institute, Kisarazu, Chiba 292-0818, Japan; Department of Applied Genomics, Kazusa DNA Research Institute, Kisarazu, Chiba 292-0818, Japan; Department of Pediatrics, Graduate School of Medicine, Gifu University, Gifu, Gifu 501-1194, Japan; Department of Applied Genomics, Kazusa DNA Research Institute, Kisarazu, Chiba 292-0818, Japan; Department of Applied Genomics, Kazusa DNA Research Institute, Kisarazu, Chiba 292-0818, Japan; Department of Medical Genetics, Shinshu University School of Medicine, Matsumoto, Nagano 390-8621, Japan; Center for Medical Genetics, Shinshu University Hospital, Matsumoto, Nagano 390-8621, Japan; Division of Clinical Sequencing, Shinshu University School of Medicine, Matsumoto, Nagano 390-8621, Japan; Department of Medical Genetics, Shinshu University School of Medicine, Matsumoto, Nagano 390-8621, Japan; Center for Medical Genetics, Shinshu University Hospital, Matsumoto, Nagano 390-8621, Japan; Division of Clinical Sequencing, Shinshu University School of Medicine, Matsumoto, Nagano 390-8621, Japan; Division of Collaborative Research, National Research Institute for Child Health and Development, Setagaya-ku, Tokyo 157-8535, Japan; Department of Molecular Endocrinology, National Research Institute for Child Health and Development, Setagaya-ku, Tokyo 157-8535, Japan; Department of Genome Medicine, National Center for Child Health and Development, Setagaya-ku, Tokyo 157-8535, Japan; Division of Collaborative Research, National Research Institute for Child Health and Development, Setagaya-ku, Tokyo 157-8535, Japan; Department of Molecular Endocrinology, National Research Institute for Child Health and Development, Setagaya-ku, Tokyo 157-8535, Japan; Organization for Research Initiative and Promotion, Tottori University, Yonago, Tottori 680-8550, Japan; Organization for Research Initiative and Promotion, Tottori University, Yonago, Tottori 680-8550, Japan; Department of Human Genetics, Yokohama City University Graduate School of Medicine, Kanazawa-ku, Yokohama, Kanagawa 236-0027, Japan; Department of Rare Disease Genomics, Yokohama City University Hospital, Yokohama, Kanagawa 236-0027, Japan; Department of Human Genetics, Yokohama City University Graduate School of Medicine, Kanazawa-ku, Yokohama, Kanagawa 236-0027, Japan; Department of Rare Disease Genomics, Yokohama City University Hospital, Yokohama, Kanagawa 236-0027, Japan; Department of Human Genetics, Yokohama City University Graduate School of Medicine, Kanazawa-ku, Yokohama, Kanagawa 236-0027, Japan; Xcoo, Inc., Bunkyo-ku, Tokyo 113-0033, Japan; Department of Applied Genomics, Kazusa DNA Research Institute, Kisarazu, Chiba 292-0818, Japan; Division of Clinical Sequencing, Shinshu University School of Medicine, Matsumoto, Nagano 390-8621, Japan

## Abstract

Next-generation DNA sequencing (NGS) in short-read mode has recently been used for genetic testing in various clinical settings. NGS data accuracy is crucial in clinical settings, and several reports regarding quality control of NGS data, primarily focusing on establishing NGS sequence read accuracy, have been published thus far. Variant calling is another critical source of NGS errors that remains unexplored at the single-nucleotide level despite its established significance. In this study, we used a machine-learning-based method to establish an exome-wide benchmark of difficult-to-sequence regions at the nucleotide-residue resolution using 10 genome sequence features based on real-world NGS data accumulated in The Genome Aggregation Database (gnomAD) of the human reference genome sequence (GRCh38/hg38). The newly acquired metric, designated the ‘UNMET score,’ along with additional lines of structural information from the human genome, allowed us to assess the sequencing challenges within the exonic region of interest using conventional short-read NGS. Thus, the UNMET score could provide a basis for addressing potential sequential errors in protein-coding exons of the human reference genome sequence GRCh38/hg38 in clinical sequencing.

## Graphical Abstract

**Graphical Abstract:**
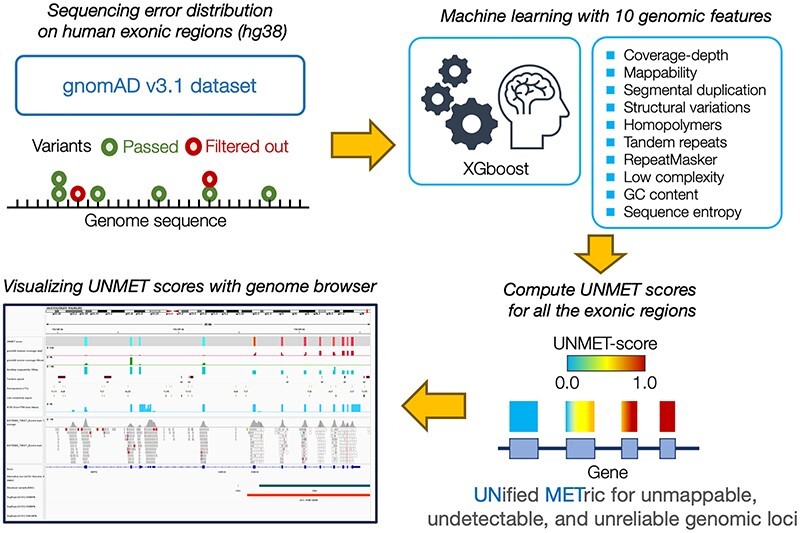

## Introduction

Nearly 20 years after its advent, massive parallel DNA sequencing, conventionally called ‘next-generation sequencing (NGS),’ has become a practical tool for analysing large volumes of DNA sequences at a reasonable cost, speed, and accuracy, and is being adopted for use in clinical diagnosis. However, using NGS for diagnostic purposes requires a benchmark to validate the accuracy of NGS-based genetic testing results. Several studies have tried to establish quality control methods to maintain high-quality NGS data and benchmarking methods for internal and external precision management of NGS systems (1–6). Furthermore, clinical genetic testing must provide appropriate caveats regarding regions with low accuracy, if any. Information on the sequencing accuracy of genetic testing must be effectively communicated to clinicians. This objective differs from benchmarking and requires providing details about the reliability of difficult-to-sequence regions at the single-nucleotide level. Following recent best practice guidelines for clinical sequencing (7), any variants detected in these unreliably sequenced regions warrant confirmation through orthogonal means. From a practical standpoint, indicating the presence or absence of these difficult-to-sequence regions within the target genes during clinical sequencing is recommended (7).

Variant calling using NGS is performed by mapping the obtained NGS reads onto the human reference genome, i.e. a ‘re-sequencing’ approach. Base calling and mapping are two different sources of errors in variant calling. The Phred score is widely regarded as the gold-standard measure of base calling accuracy, with which low-quality NGS reads are filtered out to maintain a high-quality of NGS reads. In contrast, mapping accuracy is quantitatively estimated with a mapping quality score. Nonetheless, the mapping quality score depends on both the local human reference genome sequence used to map an NGS read of interest (usually 100- to 150-nt long in the case of short-read NGS) as well as the quality of the NGS read, making it difficult to directly link the mapping quality score to variant calling error rate. The mapping quality score is reportedly affected by the presence of low-mappability regions (i.e. pseudogenes), tandem repeats, homopolymers, and other low-complexity regions (LCRs)(8). However, because difficult-to-sequence regions using NGS have been discussed in a different context in previous reports (9), quantitative measures for assessing the contribution of each sequence feature to variant calling errors in short-read NGS data have not been established.

The availability of extensive genome-wide short-read NGS data has enabled the evaluation of variant-calling error distribution within the human genome through sequencing-by-synthesis technology (4). The Genome Aggregation Database (gnomAD) v3.1 is a representative database of this kind, with data from 76 156 human genomes from unrelated individuals. This information was generated by multiple sequencing centres using short-read NGS and mapped against the human genome reference sequence. The raw data from multiple sequencing centres have been reprocessed to increase the consistency of the variant calling results across sequencing centres in the gnomAD dataset. The filter information of variants in the gnomAD dataset enables the illustration of the landscape of error-proneness of current gold-standard variant-calling methods in the human genome by short-read NGS at the nucleotide-residue resolution, which revealed a distribution of difficult-to-sequence regions via short-read NGS based on experimental data.

In this study, we generated a novel metric, termed the UNMET score, which allows the estimation of error-proneness of each nucleotide residue in the human genome using machine learning of the filtered information of variant data in gnomAD with 10 genomic sequence features. The UNMET score enables the identification of genomic regions that have a high possibility of sequence errors at the single-nucleotide level with higher sensitivity than mappability alone. Using the integrated genome viewer, the UNMET score would enable the accurate estimation of the analytical validity of genetic testing via short-read NGS, together with other lines of information, such as alternative genome sequences and/or structural changes. From a practical viewpoint, the UNMET score would significantly contribute to the reporting of appropriate caveats regarding sequencing accuracy, which is recommended by the ACMG technical standard, 2021 revision (7).

## Materials and methods

### Definition of reliable and unreliable variant call positions in gnomAD (release 3.1)

**Figure 1.**
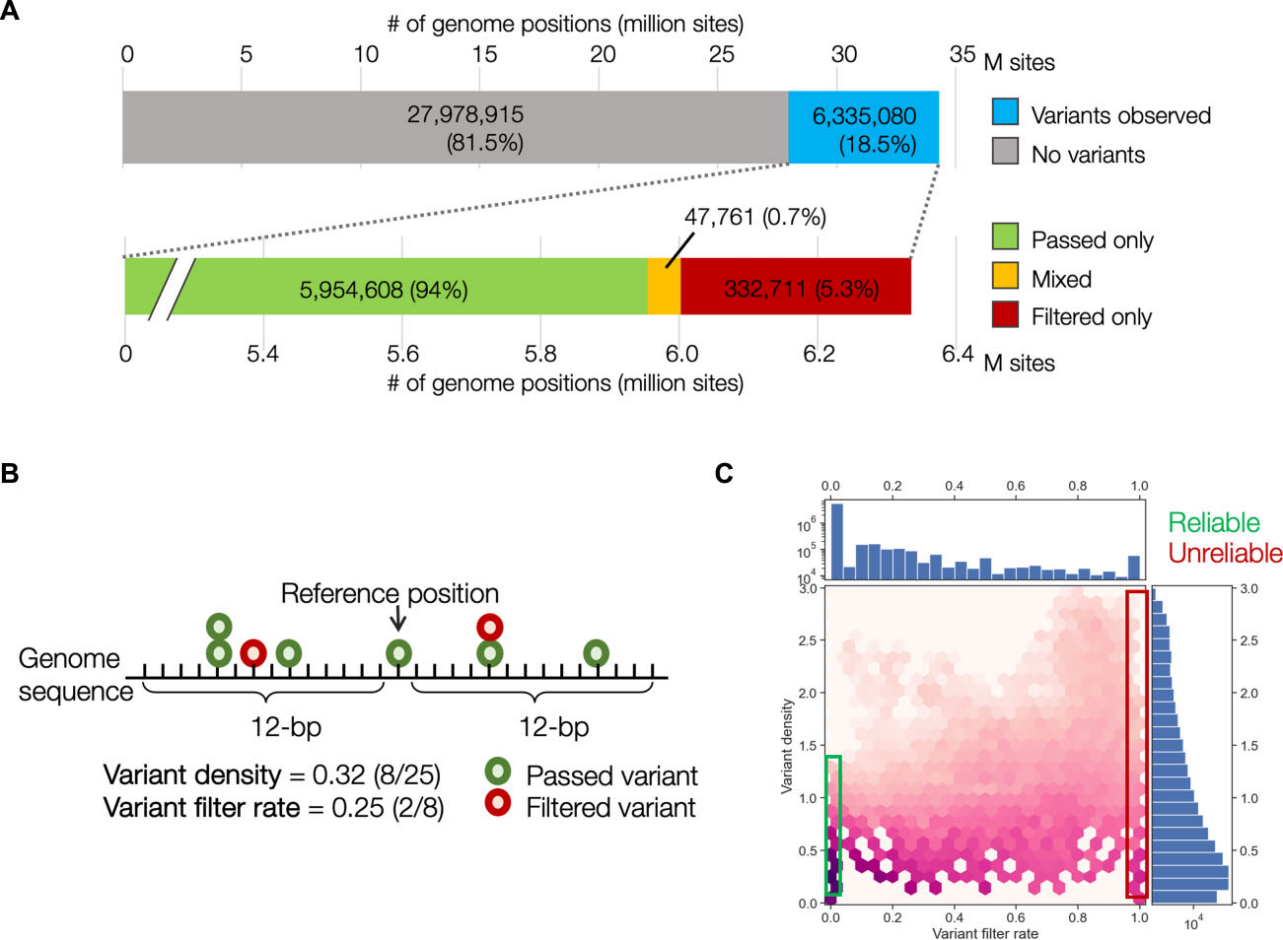
Statistics of single nucleotide variants in gnomAD version 3.1 in coding regions with filter information. **(A)** Proportion of genome positions with observed variants. **(B)** Breakdown of variant sites with filter information. Mixed indicates that two or more variants are in the same position; and both ‘passed’ and ‘filtered’ variants are observed. **(C)** Two-dimensional distributions of the variant filter rate (VFR; on the horizontal axis) and variant density (VD; on the vertical axis) for all the sites.

We used the genomic variant call data in gnomAD release 3.1 to classify whether a base position was in an error-prone region and difficult to reliably call using short-read NGS equipment, i.e. an Illumina platform. The single nucleotide variant data with the filter information in all exons with its 20-bp flanking regions in protein-coding genes were extracted from the gnomAD VCF files (downloaded from the gnomAD website, https://gnomad.broadinstitute.org/downloads). The exon and coding sequence (CDS) data were extracted from the RefSeq annotation in the human reference genome assembly GRCh38, which was obtained from NCBI Genome patch release 13 (https://www.ncbi.nlm.nih.gov/assembly/GCF_000001405.39). Within the dataset, SNVs were identified in 18.5% (6335080/34313995) of the target base positions. This implies that, theoretically, a variant was observed approximately every 5–6 base positions (Figure 1A).

To evaluate the difficulty of variant calls in genomic regions, we introduced two key indicators: variant density (VD) and variant filter rate (VFR). These indicators provide insights into the number of observed variants and the filtering process surrounding a specific variant site, thereby evaluating the reliability of variant detection at the base position.

VD is defined as the count of variants observed within an *N*-bp stretch at both ends (2*N*+ 1 bp window size). Conversely, VFR, which represents the fraction of filtered variants within a specific segment, is calculated as the number of variants filtered out within the same window divided by the total number of observed variants within that region (Figure 1B). For instance, in a segment with a 25-bp window size, 8 variants were observed, with 2 of them being filtered out. This results in a VD of 0.32 (8/25) and a VFR of 0.25 (2/8).

The values of VD and VFR depend on the number of variants within a segment of a given window size; hence, we examined how the number of variants within a segment changed as *N* varied from 2 to 24 (altering the window size from 5-bp to 49-bp). As the window size increased, the number of variants within a segment also increased. When the window size reached 25-bp, 95% of exon segments contained three or more variants (Supplementary Figure S1).

Therefore, in this study, we set *N* to 12 (resulting in a 25-bp window size). As a result, we expected to observe three or more variants in most variant sites. To evaluate the reliability of the 25-bp segment in variant detection, we examined the passed/filtered out information for other variants within the same segment.

A segment was classified as unreliable when all variants surrounding a variant site that underwent filtration were also filtered out, indicated by VFR = 1.0. Conversely, it was considered reliable when all variants within the segment surrounding a variant site that passed filters were retained, represented by VFR = 0.0.

Considering its application in machine learning, our goal was to extract a ground truth dataset for training sets involving reliable/unreliable regions. Therefore, we deemed VFR = 0.0 and 1.0 as stringent criteria for this purpose. As most segments contain three or more variants, a variant site with VD ≥ 0.12 (i.e. at least three variants observed within the 25-bp stretch) and VFR = 1.0 was classified as an unreliable variant call region. In contrast, a variant site with VD ≥ 0.12 and VFR = 0.0 was considered a reliable variant call region. The variant data within these reliable/unreliable regions were used in the machine learning process to derive the UNMET score as the ground truth dataset.

### Sequence features for machine learning

For the machine learning approach, the genomic characteristics described below were employed as features:

- *Genome coverage depth (continuous)*. The genome coverage depth of sequence reads for each base position was taken from the gnomAD data and downloaded from the gnomAD website. The coverage depth of each position was standardised by the mean and standard deviation of the coverage depth in each chromosome because of the difference in the mean of the coverage depth between autosomes and non-PARs (pseudo-autosomal regions) in the X chromosome. The absolute standardised values were used for the features.
- *Mappability (continuous)*. Genome mappability, which is a measure of how unique or repetitive regions are in the genome, for the human genome sequence was calculated using GenMap version 1.2.0 (10). The parameters of length (*K*) and errors (*E*) were set to 100 and 2, respectively. For the calculation in PAR, the sequence data of PARs in the Y-chromosome were excluded.
- *Homopolymer tract (binary)*. The homopolymer tract (a sequence of consecutive identical bases) with a stretch of 7 or more bp was extracted from the human genome sequences. The homopolymer tract segment with adjacent 12-bp flanking at both ends was considered.
- *Tandem repeat (binary)*. The tandem repeat region was sought using TandemRepeatFinder (TRF) version 4.0.9 (11), and all repeat data found were treated as tandem repeats. The region with adjacent 12-bp flanking at both ends was also considered a tandem repeat region.
- *Interspersed repeat (RepeatMasker) regions (binary)*. The interspersed repeat data were obtained from the RepeatMasker data in the UCSC genome browser (https://genome.ucsc.edu/), SINE, LINE, LTR, micro-satellites, Low complexity repeats, Satellite repeats, RNA repeats.
- *Segmental duplication (binary)*. The segmental duplications of >1000 bases of non-repeat masked sequences in the human genome were downloaded from the UCSC genome browser (https://genome.ucsc.edu/).
- *LCRs (binary)*. The LCRs were computed using the DustMasker program (12) implemented in the BLAST + 2.6.0 package (ftp://ftp.ncbi.nlm.nih.gov/blast/executables/blast+). The level option was set to 30.
- *Structural variation (binary)*. The genome regions with structural variations, including copy number variations, were obtained from the Database of Genomic Variants (DGV) (13).
- *GC content (continuous)*. The %GC content of a sequence of 25-bp in length was calculated using an in-house Python script.
- *Sequence entropy (continuous)*. The Shannon's information entropy for a nucleotide position was calculated based on the base frequencies in the adjacent sequences 25-bp in length.

### Training set

The unreliable and reliable genome positions defined above were considered the positive and negative sets, respectively, for machine learning training. The selected positions were restricted to the CDSs because of homopolymers or LCRs that are enriched in intronic regions. The number of positions for the positive and negative sets were 51 715 and 5 069 698, respectively. Because the number of positions in the negative set significantly exceeded that of the positive set, the same amount of negative data was randomly sampled from the negative set to balance the number in both sets. The dataset of 103 430 base positions with the genomic features (51 715 positive and 51 715 negative sets) were randomly split into two groups for training and testing sets in a ratio of 8:2, respectively.

### Model training

To train the model, we employed a gradient-boosted decision tree algorithm (XGBoost) (14), one of the widely used machine learning algorithms, implemented in Python (15), since the trained model showed better performance than models trained with the other machine learning algorithms, namely logistic regression and random forest, as described later. The hyperparameter set of the model (max_depth = {3, 4, 5, 6}, learning_rate = {0.1, 0.01, 0.001}, gamma = {0, 0.1, 0.01}, subsample = {0.5, 0.8, 1}, and colsample_bytree = {0.5, 0.8, 1}) was optimized using a grid search and 5-fold cross-validation with the training set. The best parameter set was selected when it led to the highest accuracy. The model was trained using the training set with the best hyperparameter set and evaluated using the test set. Model accuracy was evaluated using the area under the receiver operating characteristic curve (AUC) and Matthew's correlation coefficient (MCC). The MCC is defined as:


\begin{eqnarray*}MCC &=& ( {CPU \times CPR - PU \times PR} )/ \\ &&\sqrt {( {CPU + PU} )( {CPU + PR} )( {CPR + PU} )( {CPR + PR} )} \end{eqnarray*}

where CPU, CPR, PU and PR are the ‘correctly predicted unreliable region as unreliable,’ ‘correctly predicted reliable region as reliable,’ ‘predicted reliable region as unreliable,’ and ‘predicted unreliable region as reliable,’ respectively. The model accuracy trained with XGBoost was AUC = 0.994 and MCC = 0.964, which was higher than either that with logistic regression (AUC = 0.989 and MCC = 0.949) or with random forest (AUC = 0.993 and MCC = 0.959).

### Problematic regions for sequencing

The data for genomic regions known to cause analysis artefacts for common sequencing downstream analysis, such as alignment, variant calling, or peak calling, were obtained from the UCSC Genome Browser. The data originated from three datasets: (i) ENCODE Blacklist projects (16), which contains a comprehensive set of regions that are troublesome for high-throughput NGS aligners; (ii) Genome-in-a-Bottle project (1), which contains defined regions in which it is difficult to make a confident call due to low coverage, systematic sequencing errors, and local alignment problems and (iii) NCBI Genetic Testing Reference Materials (GeT-RM) (17), which contains highly homologous gene- and exon-level regions difficult or impossible to analyse with standard Sanger or short-read NGS approaches and are relevant to current clinical testing. Since those datasets were mapped on the genome build hg19, the genomic coordinates for the regions were converted to those in the genome build hg38 using CrossMap software (18).

### Test exome sequencing

Genomic DNA (NIST ID, HG005; RM Number, RM8393; Coriell ID, NA24631), purchased from the National Institute of Standards and Technology (Gaithersburg, MD), was used for NGS library construction using a NEXTflex Rapid DNA-Seq Kit 2.0 (PerkinElmer, Inc., Waltham, MA). Exome regions were enriched via hybridization with an IDT exome panel (Integrated DNA Technologies, Inc., Coralville, IA; The xGen Exome Research Panel v2). NGS was performed on an Illumina NextSeq2000 with a 150 nt-paired-end mode. Variants were detected using a Genome Analysis Toolkit (GATK) version 3 following the GATK Best Practices (19). The detected variants were evaluated by comparing them to the ground truth variant set using Haplotype Comparison Tools version 0.3.15 (https://github.com/Illumina/hap.py), and each variant was labelled as true positive (TP), false positive (FP), or false negative (FN).

## Results

### Derivation of the UNMET score

The accuracy of variant calling in high-quality NGS reads is determined by their mapping accuracy onto the reference genome sequence. Genome mappability, a measure of the uniqueness of regions of interest in the genome, is one of the most critical factors affecting the accuracy of variant identification via short-read NGS, but its degree of correlation with NGS accuracy is not clear (8,20). Thus, a reductionistic approach to define difficult-to-sequence regions would be inappropriate. Instead, we tried to specify difficult-to-sequence regions using an inductive approach based on experimentally accumulated NGS data as described below.

We used the gnomAD variant dataset, which consists of genome sequencing data from >76 000 people with more than 6 million single nucleotide variants (SNVs) in the coding regions with allele frequency and variant quality values. We first analysed the SNV data and its quality information in gnomAD version 3.1. To simplify the analysis, we adopted the FILTER flag (to pass or filter out data following the quality check rule of gnomAD). As the first step, we focused only on protein-coding sequences (CDS), which resulted in 34 313 995 base positions in the GRCh38 human genome annotation for further analyses (Figure 1A). Of these positions, 6 335 080 sites (18.5%) were identified to have at least one SNV regardless of the pass/filtered flags. Among the sites with variants, approximately 94% consisted of only passed variants while 5.3% contained filtered-out variants, and 0.7% contained recurrent filtered-out variants. The distribution of the VD and VFR within the 25-bp window size is shown in Figure 1C. The mean value of VD was 0.194, where 4.85 variants were observed within the flanking region, and the mean value of VFR was 0.013, indicating that an average of 1.3% of the variants observed in the same regions were filtered out. Approximately 83% of the variant sites had a VFR of 0, with no variants filtered out within both 12-bp flanking regions, indicating that the position could identify variants with high reliability. Conversely, 56 639 variant sites in CDS regions (0.8% of the variant sites) had a VFR of 1.0, indicating that all variants around these sites were filtered out, making the variants in these regions difficult to identify via short-read NGS alone. To obtain a benchmark reflecting the accuracy of variant identification using short-read NGS for each position of all CDSs at single-nucleotide resolution, we employed a machine learning approach to generate a metric designated ‘UNified METric for unmappable, undetectable, and unreliable genomic loci’ (UNMET) score for short-read NGS.

**Figure 2.**
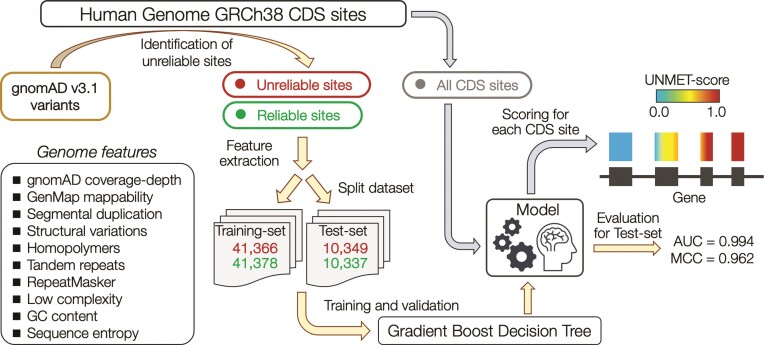
Workflow of the derivation of the UNMET score. The CDS sites on which genomic variants were located were classified into unreliable and reliable sites. Ten genomic feature vectors of the sites were used for training in machine learning to build a model that discriminates unreliable sites from reliable sites. All CDS sites were evaluated with the classifier model, and the percentile rank score of the raw score for each site was assigned as the UNMET score.

The workflow of the derivation of the UNMET score to discriminate unreliable sites from reliable sites is shown in Figure 2. To train and test the score, we selected two sets of variant sites: (i) the positive set (‘unreliable sites’), which consisted of 51 715 variant sites with a VFR = 1.0 and VD ≥ 0.12. These sites had at least three variants observed within the 25-bp stretches of the sequence, and none of the variants passed the quality control filters. (ii) The negative set (‘reliable sites’), which comprised an equal number of randomly selected variant sites with a VFR = 0.0 and VD ≥ 0.12. We did not observe any difference in the proportion of global minor allele frequency (gMAF) of the variants between the positive and negative sets (Supplementary Table S1). We randomly split the dataset into 80% for training and 20% for testing the machine learning protocol. We extracted 10 sequence-based features, such as genome mappability, homopolymers, tandem-repeat information as well as the standardised genome coverage depth in gnomAD data as the feature vectors (see Materials and methods for the details; Figure 2 and Supplementary Figure S2A). We implemented a gradient boost decision tree algorithm, XGBoost (14), to train the model to discriminate between unreliable and reliable sites. Eventually, by evaluating the model with the testing set, we observed that the classifier could split the sites with high accuracy (AUC and MCC of 0.994 and 0.962, respectively; Supplementary Figure S2B). The most important feature of the model was mappability (0.44), followed by segmental duplication (0.25), genome coverage depth of gnomAD data (0.18), and tandem repeat (0.07) (Table 1).

**Table 1.** The feature importance of the XGBoost model

Feature	Importance
GenMap mappability	0.444
Segmental duplication	0.250
gnomAD coverage-depth	0.177
Tandem repeats	0.068
Structural variations	0.023
Homopolymers	0.019
GC content	0.007
Low complexity	0.006
Sequence entropy	0.003
RepeatMasker	0.003

To evaluate the contribution of individual sequence features to the performance of discrimination, we performed a univariate analysis. Among the single features, genome coverage depth and mappability showed the best performance, followed by segmental duplication and structural variation features (Supplementary Table S2). This shows that these features are highly correlated with each other (Supplementary Figure S2). The remaining six features, including tandem repeat, homopolymer tract, and %GC content, showed poor performance. Nevertheless, none of the single features outperformed the model trained with all the features.

Because highly repetitive sequences were frequently included in the regions of low mappability, the 25-bp stretches around the variant sites with high sequence similarity might have been contained in both the training and test sets, which may have caused information leakage in the training process. Thus, we verified this using a test set that excluded variant sites with 80% or more sequence similarity within the 25-bp stretches compared to those in the training set (Supplementary Table S3). The AUC and MCC of the test set were 0.991 and 0.952, respectively. The slightly lower accuracy metrics indicated that information leakage due to sequence similarity around the sites was minimal.

We then applied the prediction model to all CDS positions to assign the prediction scores, which were converted to normalised percentile rank scores, defined as UNMET scores, with a value close to 1.0, indicating that the base position was unfavourable for accurately identifying variants with high confidence.

### Evaluation of the reliability of the UNMET score

Next, we evaluated the reliability of the UNMET score by comparisons with established measurement scores for the quality of each variant or dataset in genomic regions known to be difficult for sequencing or detecting genome variants.

#### 1. Allele-specific variant quality score log-odds (AS_VQSLOD)

**Figure 3.**
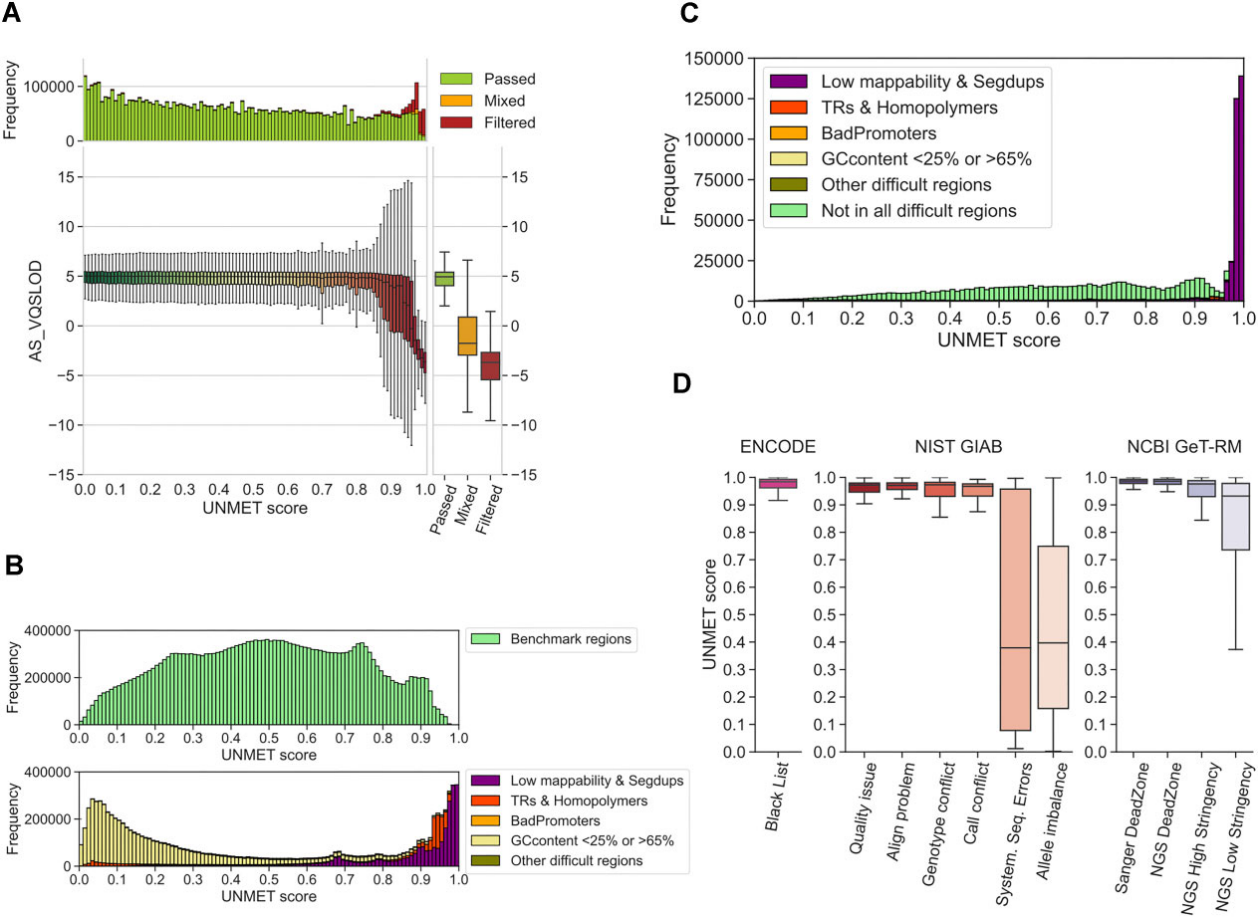
Evaluation of the reliability of the UNMET score. **(A)** 2D distribution plot of the UNMET score (x-axis) and AS_VQSLOD score (y-axis). The main panel depicts boxplots of AS_VQSLOD distributions in each UNMET score bin. **(B)** UNMET distribution in the benchmark (upper) and difficult regions designated by the GIAB dataset (lower). The datasets are as follows: Low mappability & Segmental duplications, regions with low mappability or segmental duplications; TRs & Homopolymers, merged all tandem repeats and homopolymers with 5-bp flanking regions; BadPromoters, transcription start sites or first exons that have systematically low coverage; Other difficult regions, miscellaneous difficult regions, including highly variable copy numbers in the population, such as major histcompatibility complex (MHC), T-cell and B-cell receptors, and killer-cell immunoglobulin-like receptors (KIRs); GCcontent <25% or >65%, %GC content < 25% or >65% with 50-bp flanking regions. **(C)** UNMET distribution in CDS position with variant density = 0. **(D)** UNMET distribution in the stratified problematic regions for NGS in the six datasets from the ENCODE Blacklist (16), GIAB (22) and NCBI GeT-RM (17), which were implemented in the UCSC Genome Browser (https://genome.ucsc.edu/).

For evaluating the reliability of the UNMET score, we first compared the UNMET scores for each genomic position to the other measurements for variant quality. First, we compared the UNMET score to the AS_VQSLOD score, a variant quality measurement adopted in gnomAD v3. As expected, the UNMET score displayed an inverse correlation to the AS_VQSLOD score (Figure 3A). The AS_VQSLOD values decreased as the UNMET score reached close to 1, and most variants with an UNMET score >0.98 were filtered out, whereas most variants at positions with an UNMET score <0.90 had passed, suggesting that the CDS positions with a high UNMET score, especially >0.97, were unreliable for genomic variant identification using short-read NGS.

#### 2. The genome in a bottle consortium (GIAB) benchmark regions

We analysed the distribution of UNMET scores in the CDS positions inside the benchmark and difficult-to-sequence regions in the human reference genome DNA described previously (1,3). We compared the distribution of UNMET scores in the benchmark regions provided by GIAB with the difficult regions where genomic variants are not reliably identified owing to technical difficulties, such as repetitive sequences (Figure 3B). The UNMET scores in the benchmark regions were broadly distributed but most were <0.96. Notably, the score in the difficult regions showed a bimodal distribution, and one of the peaks had an UNMET score between 0.98 and 1.0, indicating that the score reasonably captured the genomic regions where false or misidentified genomic variants were prevalent. However, the other peak skewed towards lower UNMET scores for these difficult regions.

To further explore these variations, we examined the UNMET score distributions within each stratification of GIAB difficult regions, including regions with: (i) low mappability and segmental duplication, (ii) tandem repeats and homopolymers, (iii) bad promoters, (iv) other difficult regions and (v) %GC content <25% or >65% (Figure 3B). Notably, the distribution within low mappability or repeat regions was biased towards higher UNMET scores, indicating reliability in these regions. In contrast, regions with low and high %GC content were skewed towards the lower side. In line with the gnomAD dataset, the frequently filtered-out variants were not enriched in the regions with low and high %GC content compared to those with low mappability or repeats (Supplementary Figure S3). This indicates that these regions may not be inherently unreliable for variant identification. Although low and high %GC regions are known to cause underrepresented sequencing reads/counts in short-read NGS (21), our results indicate that they do not substantially affect the sequencing error rate. Therefore, it appears reasonable to include these regions within benchmark areas, rather than categorising them as difficult regions.

#### 3. No variant detected regions in gnomAD

Third, we analysed the CDS positions with a VD = 0 (956 205 base positions in approximately 2.7% of all the CDS positions), which denotes positions with no identified SNVs in the gnomAD v3.1 dataset. There are three possible explanations for regions to have a VD = 0: first, the region could be essential for some biological functions, making its sequence relatively conserved. Second, a larger population size (>76 000) could be required to observe variants in these regions. Third, SNVs in these regions could be completely undetectable using short-read NGS. All three situations may occur in real-world data. As per the definition, VFR could not be calculated for regions with VD = 0; therefore, these regions were not included in the training set. We observed a skewed UNMET score distribution toward higher scores in the VD = 0 regions (Figure 3C), with ∼30% of positions having an UNMET score > 0.96 and consistently overlapping to low mappability and segmental duplication, suggesting that variants in these regions could not be properly identified owing to technical reasons. However, without information on VD = 0 regions, the training set may underestimate regions with lower UNMET scores.

#### 4. Previously reported ‘difficult-to-sequence’ regions

We analysed the distribution of UNMET scores at nucleotide residues in regions previously reported as problematic for NGS or Sanger sequencing. These regions were provided by the UCSC Genome Browser (https://genome.ucsc.edu/), including ENCODE Blacklist (16), NIST GIAB (22), and NCBI GeT-RM (17) (Figure 3D). Among the problematic regions, the median values of the UNMET scores ranged around 0.97, indicating that this value was a threshold of error-proneness. Although the classification of GIAB benchmark regions and difficult regions did not illustrate a sharp discrimination of UNMET scores, it was consistent with the threshold of the determined UNMET score (Figure 3B). The genomic regions longer than 50-nt contiguous bases with an UNMET score ≥0.97 are listed in Supplementary Table S4, which can be considered a list of ‘difficult-to-sequence’ regions. The list consists of 14121 CDS exons from 1133 genes, and of those, 147 were disease-associated genes (Supplementary Table S4).

#### 5. Indel variant sites in gnomAD

**Figure 4.**
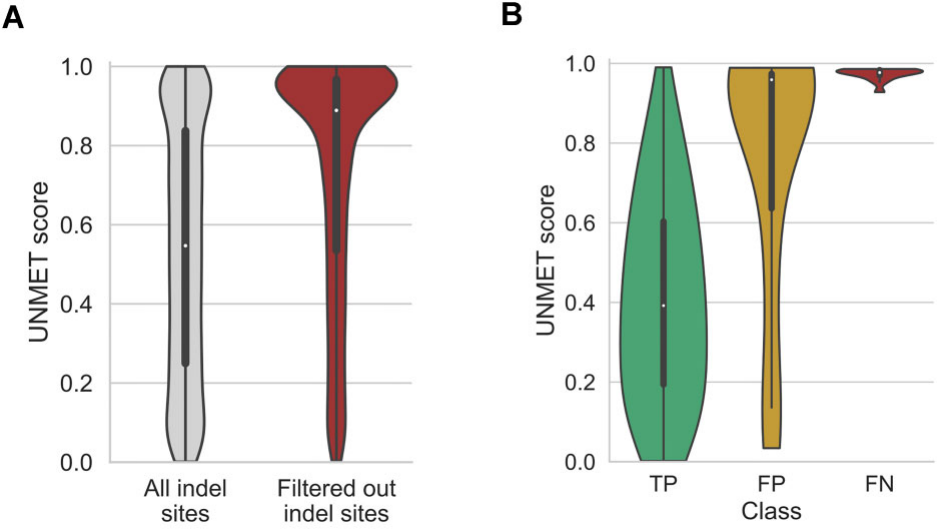
**(A)** Distribution of UNMET scores on indel sites. All the indels in CDS sites were observed in the gnomAD v3.1 database (left), and two or more indels were observed but all of them were filtered out (right). **(B)** Practical evaluation of the UNMET score with exome sequencing data of the platinum genome sample. The violin plots display the distributions of the UNMET scores for sites with true positive (TP), false positive (FP) and false negative (FN) variants, respectively, when those sites were compared with the ground truth variants in the GIAB benchmark set.

Next, we focused on CDS positions with small insertion/deletion variants in the gnomAD dataset because the indel variants were also expected to affect the mapping quality of the short reads. We identified 336 812 indel variant sites in the CDS regions, regardless of whether they were passed or filtered out, and 7955 sites out of those had two or more indel variants filtered out (Figure 4A). The mean UNMET score in the sites with recurrently filtered out indel variants (0.73 S.D. ± 0.30) was significantly higher than that in the remaining indel variant sites (0.53 S.D. ± 0.31; *t*-test, *P*-value < 1.0 × 10^−300^), indicating that the UNMET score could also discriminate the CDS positions with falsely detected indel variants.

#### 6. Real exome data of GIAB reference genome (HG005)

We further evaluated the UNMET scores for real exome sequencing of an individual sample and observed that it mimicked a practical clinical sequencing situation. We used an updated short-read NGS (NextSeq2000, Illumina) because the gnomAD data were an aggregation of different sequencing conditions by different sequencing centres. Since the recent short-read NGS operates in a 2-colour mode, it is important to consider the possibility of a transition in detection fluorescent colour mode, as the previous NGS system used a 4-colour mode. Thus, we performed exome sequencing of one of the individual samples provided by the GIAB project designated as HG005 (Coriell ID, NA24631; NIST RM Number, RM8393), and the genomic variants were identified using a standard protocol assisted by the GATK v3 variant caller also used in the gnomAD dataset. We then verified the variants observed in the GIAB benchmark region with the variants confidently detected using various sequencing methodologies (22).

Following the reported variant data of HG005 (23), the ground truth variants in the benchmark region consisted of 6913 homo- and 10 073 hetero-SNVs. We compared the SNVs that we identified independently using the ground truth SNV data and labelled them as TP, FP, and FN. For homo-SNVs, most of the variants exhibited high QUAL values and were correctly detected in our exome sequencing (Supplementary Figure S4 and Supplementary Table S5). However, the QUAL values were slightly lower for hetero-SNVs than those for homo-SNVs, and the numbers of false-positive (209 SNVs) and false-negative variants (14 SNVs) were increased, indicating that the base positions with FP and FN variants were in error-prone sequence regions. The distribution of UNMET scores for the sites with heterozygous SNVs was significantly different among the TP, FP, and FN variants (Figure 4B). Notably, the sites of FP variants with high QUAL values also showed high UNMET scores. These results demonstrated that the UNMET score could predict the variant calling accuracy for each CDS position in the updated practical settings of exome sequencing.

### Visualization of UNMET scores for evaluation of ‘error-proneness’ of genomic residues in the exome regions for clinical NGS applications

**Figure 5.**
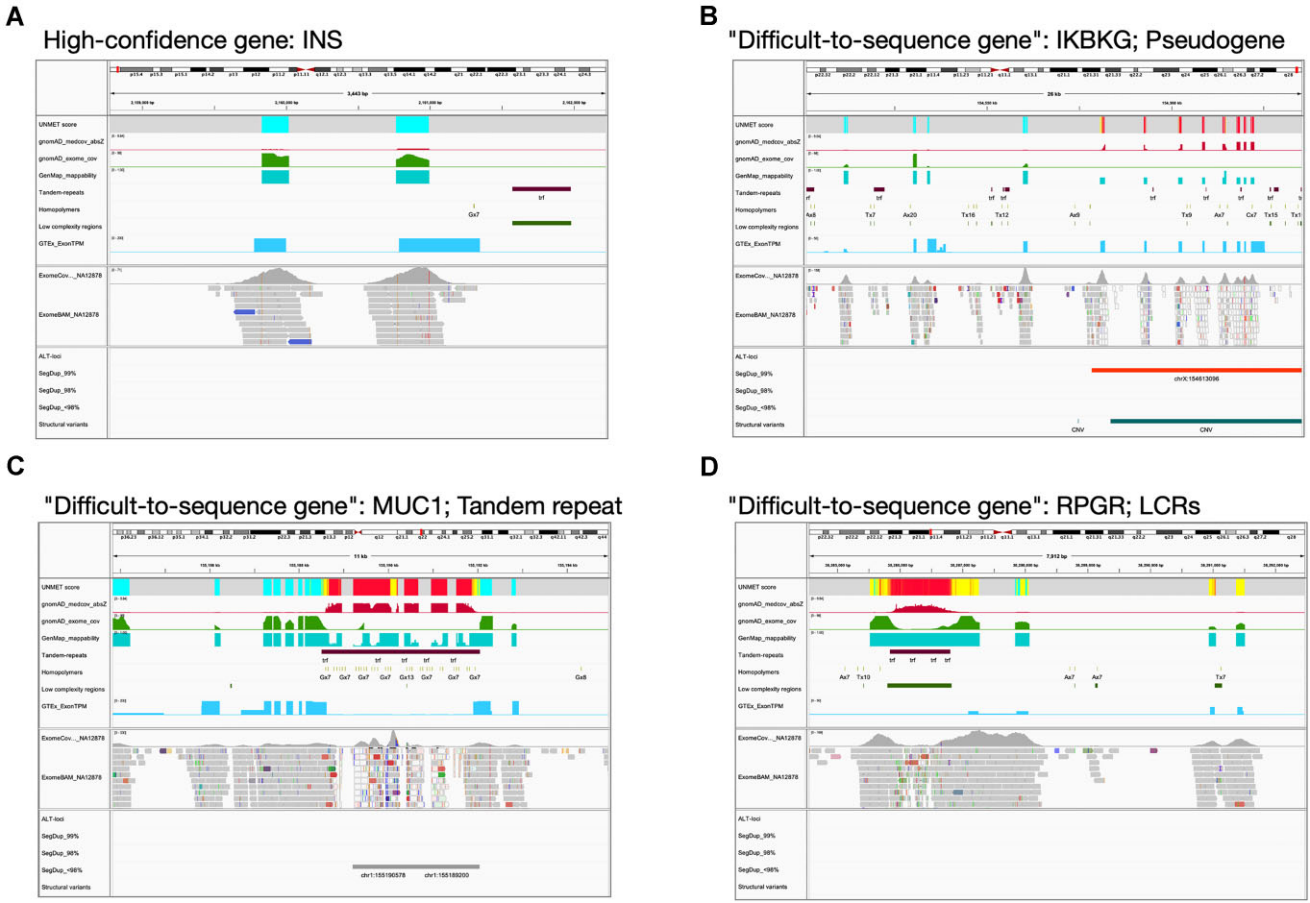
Examples showing the visualization of ‘difficult-to-sequence’ genes on IGV. All data required for visualization on IGV as well as the session file are available from Zenodo: https://doi.org/10.5281/zenodo.10061054, aside from actual NGS exome data, which are included for confirmation of the consistency of the UNMET score with the actual NGS data under the running conditions employed at each laboratory. Panels exhibit examples of high-confidence genes (*IDS*, panel A) and ‘difficult-to-sequence’ genes (panel B, *IKBKG*; panel C, *MUC1*; panel D, *RPGR*).

The UNMET score does not directly indicate sequencing error rates like the Phred value; thus, we classified the UNMET score into ‘difficult-to-sequence,’ ‘error-prone,’ and ‘reliable’ regions via short-read NGS as described above. The UNMET score is defined as a normalised percentile rank score and thus cannot offer a clear boundary between error-prone and reliable residues. To provide a quantitative rationale, we compared the distribution of UNMET scores in two datasets. The first dataset includes regions from the NGS DeadZone dataset provided by Mandelker et al. (24), where short reads cannot be unambiguously mapped to any positions in an exon or large stretches considered to be ‘highly error-prone.’ The second dataset encompasses the GIAB benchmark regions, where all variants are expected to be reliably detected. Approximately 85% of the base positions in NGS DeadZone had an UNMET score ≥0.97, while 99% of the base positions in the benchmark regions had an UNMET score <0.93. Considering this situation, we visualized the UNMET score as a heat map, where difficult-to-sequence (≥0.97), error-prone (0.93–0.97), and reliable (<0.93) residues are shown in red, yellow, and green, respectively, using Integrated Genomics Viewer (IGV, Figure 5) (25,26). In IGV, we also show several lines of information on genomic features together with the heat map of the UNMET score for helping users understand the reason why the region is scored as such: (i) gnomAD median coverage absolute z-value, (ii) gnomAD exome coverage (lift over from hg19), (iii) GenMap mappability (window size, 150 nucleotides long; max number of mismatches, 2), (iv) tandem repeat, (v) homopolymeric region (>7×), (vi) LCRs and (vii) gene expression level in each exon obtained from the GTEx database (https://gtexportal.org/), which might improve rare variant interpretation (27). Additionally, we recommend visualizing actual exome data obtained by each laboratory to confirm the consistency of the UNMET score with actual data under updated NGS conditions. For example, the exome data obtained in our NGS system (GIAB reference genome DNA, HG005, sequenced on a NextSeq2000 (Illumina)) are also presented, as shown in the middle columns of Figure 5. In the bottom part of Figure 5, the following lines of information are given as supplements: exon–intron structure of genes, alternative loci, structural variants, and segmental duplications (classified by sequence similarity). Because a part of the information delivered by the bottom part is not reflected on the UNMET score but is useful for considering the analytical validity of genes of interest via short-read NGS, we consider it highly informative to obtain these lines of information in a one-stop snapshot: structural variations, including recombination, duplication, and large deletion, that could cause diseases in some cases although UNMET scores derived from short-read NGS data of healthy donors do not inform about these possibilities.

Figure 5 demonstrates typical examples of visualization of analytical validity of genes that contain highly homologous regions to pseudogenes (Figure 5B, for *IKBKG*), tandem repeats (Figure 5C, for *MUC1*), or LCRs (Figure 5D, for *RPGR*). These results indicate that this visualization tool makes it easy to overview the landscape of the error-prone regions at a glance and helps understand the cause of sequencing difficulty.

## Discussion

gnomAD is an indispensable data resource for human genetics, produced by the aggregation and harmonization of variant calls made by a well-controlled bioinformatics pipeline from a variety of large-scale sequencing projects. The variant data of gnomAD v3.1 was accumulated from 76156 genomes from unrelated individuals and generated using multiple short-read sequencing platforms at different sequencing centres. Thus, using variant calling data from gnomAD v3.1 was suitable for the evaluation of intrinsic variant calling errors, considering the local genome sequences and polymorphic nature of private genomes. This approach helps minimize the contribution of accidental NGS technical issues, as the extensive accumulation of data in gnomAD reduces their contribution. Although the occurrence of NGS errors has been vaguely explained by linking several plausible causes (mapping errors and specific features of genome sequence, etc.), the gnomAD dataset enabled us to draw a solid picture of NGS error distribution via short-read NGS of the genome and sequence feature effects on NGS accuracy.

When utilising NGS for diagnostic purposes, it is common to assess its analytical validity specifically for the genomic regions of interest. To address this, we devised the UNMET score, which is derived through machine learning using real-world human NGS data from over 70 000 healthy individuals in gnomAD v3.1. However, it is important to note that the UNMET score is considerably dependent on the version of the reference human genome sequence, aside from NGS running conditions (e.g. read length and DNA insert size in the library). Several variant-calling discrepancies of exome sequencing exist owing to reference genome differences between GRCh37/hg19 and GRCh38/hg38 (28). Moreover, both reference genome sequences contain false duplications even in medically relevant genes as clarified by analysis of haplotype-resolved whole genome assembly of the HG002 genome (29). Because the training and validation data of gnomAD used in this study was based on the human reference genome version GRCh38/hg38, the current UNMET score is specific for this version of the human reference genome. Thus, UNMET scores for the false-duplicated regions in each genome assembly must be re-evaluated using an appropriate genome sequence as a reference. If we accept the small uncertainty of reliability of the obtained UNMET score, we can calculate the UNMET score from only 10 genome sequence features using machine learning data in this study. Another limitation is that we focused specifically on protein-coding exons, despite whole genome sequencing gaining popularity. An UNMET score for whole genome sequencing would require a new training dataset for machine learning because the intergenic and intronic sequence features are quite different from those in the coding regions.

Accurate clinical applications of NGS gene testing would require the identification of unexplored or undetermined regions by NGS in advance. However, because of the broad target sequences to be tested, it is difficult for clinicians and patients to estimate the missing regions by NGS in genetic testing. The UNMET score would make it easy to identify difficult-to-sequence regions in a gene of interest and thus judge whether we should carry out additional ancillary analyses to confirm the sequences of these regions. In conclusion, the UNMET score can serve as a reliable measure for assessing the sequencing validity of genetic testing through short-read NGS at the single-nucleotide resolution.

## Supplementary Material

gkad1140_Supplemental_Files

## Data Availability

The UNMET score mapping data can be visualized in Integrated Genome Viewer (IGV), and the session file is freely available from Zenodo: https://doi.org/10.5281/zenodo.10061054. The exome sequencing data of human reference genome DNA (HG005) were deposited to DDBJ Sequence Read Archive (DRA, https://www.ddbj.nig.ac.jp/dra/index.html) under the run accession ID of DRR415794 (Submission Accession ID, DRA015095).
