# Supplementary material for: Exome-wide benchmark of difficult-to-sequence regions using short-read next-generation DNA sequencing
Source: Nucleic Acids Res. 2023 Nov 28;52(1):114–24. doi: 10.1093/nar/gkad1140 (PMC10783491; doi:10.1093/nar/gkad1140)
Supplement: gkad1140_Supplemental_Files [file gkad1140_supplemental_files.zip › Hijikata_etal_SupplFigures.docx]

**Supplementary Figures**

**Figure S1.** Distribution of the number of observed variants within a particular window size of variant position. The vertical dotted line indicates the cutoff value for two or more variants observed in the window. The horizontal dotted line indicates that 95% coverage of positions in which more than X variants were observed. With a window size of 25 bp, the number of positions with two or more other variants in the same window size reached approximately 95% of all the positions.


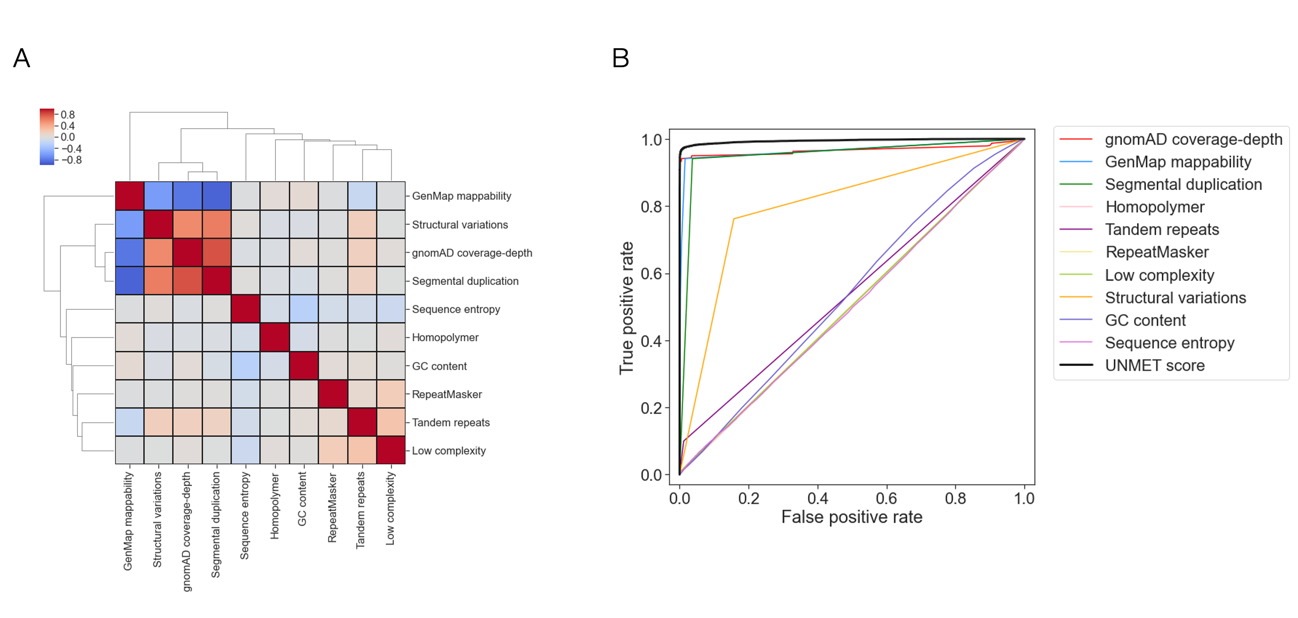


**Figure S2**. Genomic features and the use of machine learning for calculating UNMET score. (A) Heatmap of the Spearman’s correlation coefficients between the 10 genomic features used for machine learning. (B) Receiver operating characteristic (ROC) curves for evaluating the prediction accuracy. UNMET score (black) indicates the evaluation results of training with all of the features.

**Figure S3.** Distribution of UNMET score in each stratified difficult region in the GIAB datasets. The datasets are as follows: Low mappability & Segmental duplications, regions with low mappability or segmental duplications; TRs & Homopolymers, merged all tandem repeats and homopolymers with 5-bp flanking regions; BadPromoters, transcription start sites or first exons that have systematically low coverage; Other difficult regions, miscellaneous difficult regions, including highly variable in copy number in the population such as major histcompatibility complex (MHC), T-cell and B-cell receptors, and killer-cell immunoglobulin-like receptors (KIRs); GCcontent <25% or >65%, %GC contents lower than 25% or greater than 65% with 50-bp flanking regions; Not in all difficult regions, excluded all the regions described above.

**Figure S4.** Proportion of each bin of the variant filter rate (VFR) in 25-bp window size for base positions in the stratified difficult regions and non-difficult regions in the GIAB dataset.


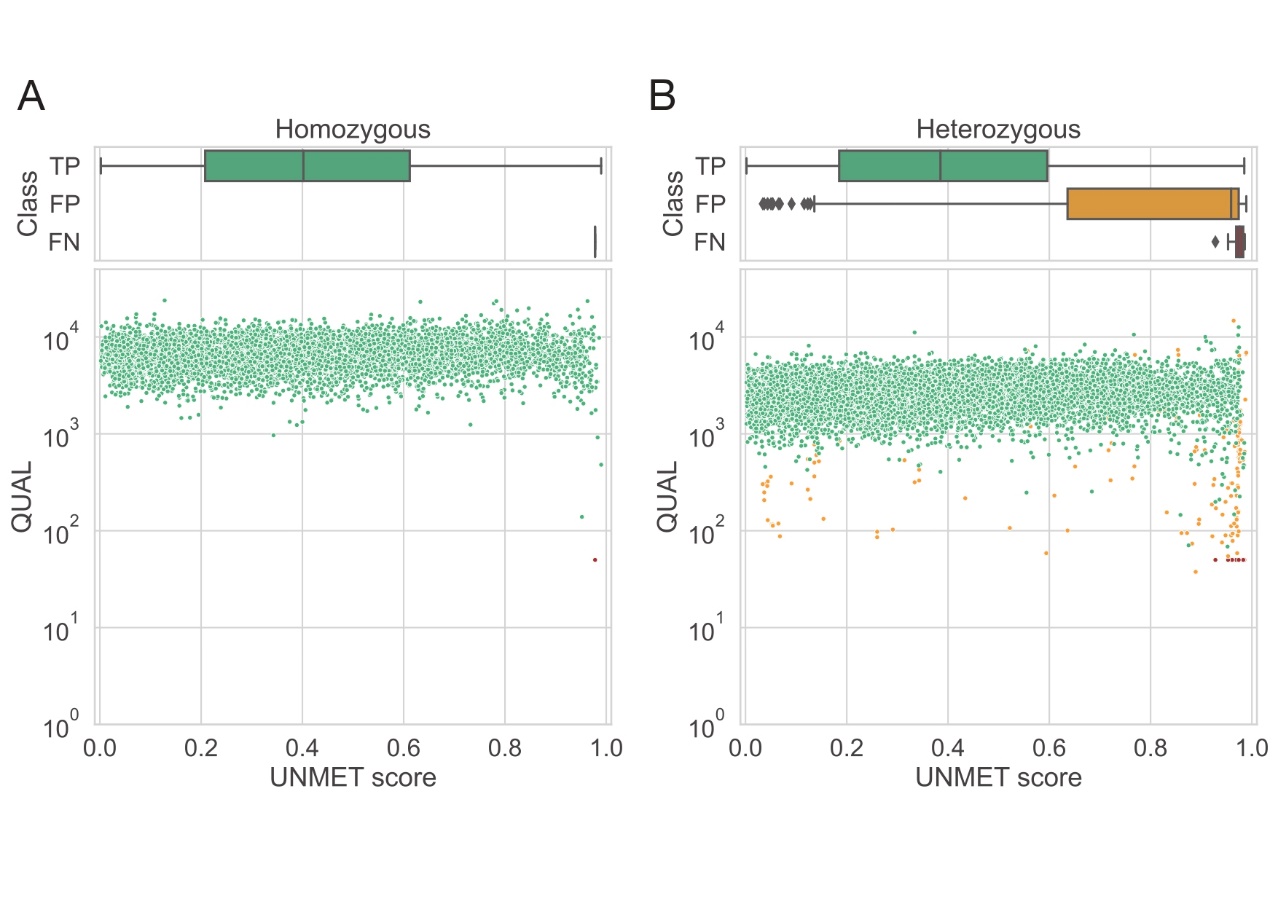


**Figure S5.** Practical evaluation of the UNMET score with exome sequencing data of the platinum genome sample. Two-dimensional distribution plot of the UNMET score (x-axis) and QUAL score (y-axis) for (A) homozygous and (B) heterozygous variant sites.
